# A Novel UC Exclusion Diet and Antibiotics for Treatment of Mild to Moderate Pediatric Ulcerative Colitis: A Prospective Open-Label Pilot Study

**DOI:** 10.3390/nu13113736

**Published:** 2021-10-23

**Authors:** Chen Sarbagili-Shabat, Lindsey Albenberg, Johan Van Limbergen, Naomi Pressman, Anthony Otley, Michal Yaakov, Eytan Wine, Dror Weiner, Arie Levine

**Affiliations:** 1 PIBD Research Center, Paediatric Gastroenterology and Nutrition Unit, The E. Wolfson Medical Center, Holon 5822012, Israel; ibd.chen@gmail.com (C.S.-S.); michal.yaakov@walla.co.il (M.Y.); drorweiner@gmail.com (D.W.); 2 The Sackler Faculty of Medicine, Tel-Aviv University, Tel-Aviv 6997801, Israel; 3 Division of Gastroenterology, Hepatology, and Nutrition, Children’s Hospital of Philadelphia, Philadelphia, PA 19104, USA; AlbenbergL@chop.edu (L.A.); PressmanN@chop.edu (N.P.); 4 Division of Pediatric Gastroenterology and Nutrition, Department of Pediatrics, Amsterdam University Medical Centers, Emma Children’s Hospital, 1105 AZ Amsterdam, The Netherlands; j.e.vanlimbergen@amsterdamumc.nl; 5 Tytgat Institute for Liver and Intestinal Research, Amsterdam Gastroenterology Endocrinology and Metabolism, Academic Medical Center, University of Amsterdam, 1105 BK Amsterdam, The Netherlands; 6 Division of Gastroenterology and Nutrition, IWK Health Center, Halifax, NS B3K 6R8, Canada; Anthony.otley@dal.ca; 7 Departments of Pediatrics and Physiology, University of Alberta, Edmonton, AB T6G 2R3, Canada; wine@ualberta.ca

**Keywords:** ulcerative colitis, child, diet, antibiotics, remission, treatment

## Abstract

Background: As the microbiome plays an important role in instigating inflammation in ulcerative colitis (UC), strategies targeting the microbiome may offer an alternative therapeutic approach. The goal of the pilot trial was to evaluate the potential efficacy and feasibility of a novel UC exclusion diet (UCED) for clinical remission, as well as the potential of sequential antibiotics for diet-refractory patients to achieve remission without steroids. Methods: This was a prospective, single-arm, multicenter, open-label pilot study in patients aged 8–19, with pediatric UC activity index (PUCAI) scores >10 on stable maintenance therapy. Patients failing to enter remission (PUCAI < 10) on the diet could receive a 14-day course of amoxycillin, metronidazole and doxycycline (AMD), and were re-assessed on day 21. The primary endpoint was intention-to-treat (ITT) remission at week 6, with UCED as the only intervention. Results: Twenty-four UCED treatment courses were given to 23 eligible children (mean age: 15.3 ± 2.9 years). The median PUCAI decreased from 35 (30–40) at baseline to 12.5 (5–30) at week 6 (*p* = 0.001). Clinical remission with UCED alone was achieved in 9/24 (37.5%). The median fecal calprotectin declined from 818 (630.0–1880.0) μg/g at baseline to 592.0 (140.7–1555.0) μg/g at week 6 (*p* > 0.05). Eight patients received treatment with antibiotics after failing on the diet; 4/8 (50.0%) subsequently entered remission 3 weeks later. Conclusion: The UCED appears to be effective and feasible for the induction of remission in children with mild to moderate UC. The sequential use of UCED followed by antibiotic therapy needs to be evaluated as a microbiome-targeted, steroid-sparing strategy.

## 1. Introduction

Ulcerative colitis (UC) in children is a chronic inflammatory disorder of the colon, associated with clinical symptoms including diarrhea and rectal bleeding, and has a negative impact on quality of life [1]. Epidemiologic studies have demonstrated an overall increase in the prevalence and the incidence of UC in both developed and developing countries [2,3]. The sharp change in food consumption from a non-Western to a Western diet has been suggested to contribute to this trend [3,4]. The pathogenesis of ulcerative colitis (UC) is believed to be related to dysbiosis coupled with diminished host-barrier function and unrepressed inflammation. The dysbiosis in UC is characterized by a reduction in short-chain-fatty-acid (SCFA)-producing taxa [5,6] and, in some studies, by an increase in potential pathobionts, such as *Escherichia* or *Ruminococcus gnavus* [7], or hydrogen-sulfide-reducing bacteria [6]. The manipulation of the microbiota has become one of the most intriguing targets for intervention in inflammatory bowel diseases (IBDs). Fecal microbiota transplantation (FMT), a straightforward therapy that manipulates the microbiota, has been shown to be effective in the short term in about 30% of cases [8]. The success of FMT appears to depend upon the choice of donor and their microbiota composition [9]. Recent data suggest that diet may alter intestinal microbiota, directly affect the host epithelial and goblet cells, diminish antimicrobial peptides, and influence the immune system’s responses [4,10,11,12,13,14]. However, while dietary interventions have proven to be highly effective in inducing remission in Crohn’s disease, the role of diet in UC and the potential for dietary therapies remain elusive [15,16,17]. A recent guideline from the international organization for the study of inflammatory bowel disease (IOIBD), primarily based on epidemiologic and animal models, suggested that patients with UC should reduce exposure to red or processed meat; saturated, trans and dairy fat; and certain additives [18]. However, there are no prospective randomized controlled trials with dietary interventions published to date that have demonstrated that a dietary intervention can induce remission in active UC in children. Currently, the only non-biologic medication for UC, which does not suppress the immune system, is 5-aminosalicylic acid (5ASA), which is considered to be the first-line therapy in mild to moderate cases. However, medications such as steroids, immunomodulators (IMM) and biologics have been increasingly used in the treatment of pediatric UC.

The manipulation of the intestinal microbiome is an emerging new strategy for the treatment of IBD, which may reduce the need for immunosuppression. Two strategies that might alter the microbiome and could be used in conjunction are diet and antibiotics. Dietary components can modulate the composition and metabolome of the gut microbiota, as well as affect the intestinal epithelium, goblet cells and innate immune system [12]. Dietary factors present in Western diets may decrease the production of mucins or cause more permeable mucous and antimicrobial peptides, as well as reshaping the microbiota [13,19]. Turner et al. demonstrated that patients with severe UC have an increased relative abundance of *Gammaproteobacteria* [7]. The presence of pathobionts may make a disease amenable to antibiotic therapy. Antibiotic combinations have been used as a microbiota-targeted therapy, including a triple combination of amoxicillin, tetracycline and metronidazole, and have been shown to be effective in active adult UC patients [20,21].

We hypothesize that the UC disease course can be controlled either by using a novel diet, developed especially for the induction of remission in UC; by an antibiotic strategy; or by both. To test the feasibility and efficacy of the diet, we decided to examine these strategies in a pilot trial in children with mild to moderate UC.

## 2. Materials and Methods

### 2.1. Study Population and Design

**Figure 1:**
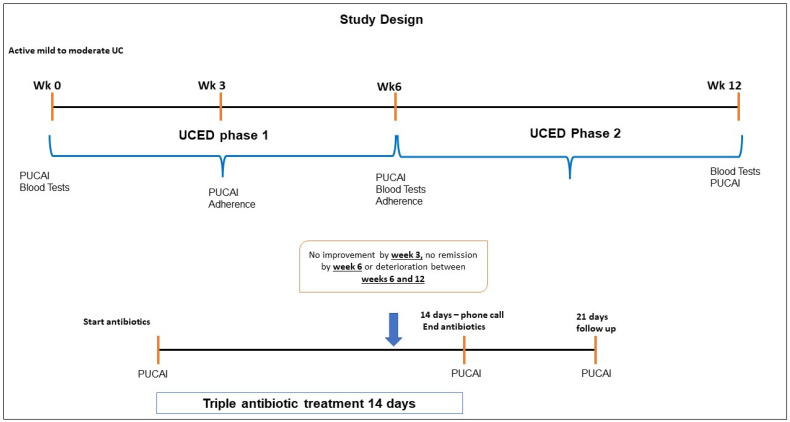
Trial design.

This was an open-label, prospective, single-arm, multicenter pilot study involving the treatment of children with active mild to moderate UC using a novel diet with an antibiotic rescue design for dietary failures. The study population targeted patients aged 8–19, with mild to moderate active disease, defined according to the Pediatric UC Activity Index (10 ≤ PUCAI ≤ 45), at diagnosis or despite maintenance therapy with 5ASA or thiopurines, stable for at least 6 weeks. All the recruited patients meeting the inclusion and exclusion criteria (see below) were introduced to a novel dietary intervention (Supplementary Table S1) termed the Ulcerative Colitis Exclusion Diet (UCED), for the first 6 weeks, and those in remission at week 6 received a step-down diet for another 6 weeks. Patients with no improvement by week 3, who failed to achieve remission by week 6, or who deteriorated between weeks 6 and 12 could decide to receive a 14-day course of amoxicillin (50 mg/kg/day; max: 500 mg of TID), metronidazole (15 mg/kg/day; max: 250 mg of TID) and doxycycline (4 mg/kg/day; max: 100 mg of BID) (AMD). Subjects with intolerance to AMD were instructed to discontinue the metronidazole. The patients on AMD were seen again 7 days after the cessation of antibiotics (Figure 1). The patients were instructed to continue their pretreatment (5ASA or immunomodulators) without any dose change. The primary outcome was the clinical remission rate (PUCAI < 10) at week 6 for the UCED intervention. The study took place at 3 sites: the Wolfson Medical Center, Holon, Israel; the Children’s Hospital of Philadelphia, PA, USA; and the IWK Health Center, Halifax, NS, Canada. All the patients signed informed consent, and all the sites obtained ethical approval (NCT 02345733).

### 2.2. The Ulcerative Colitis Diet Intervention

The UCED diet was designed to alter dietary components that may adversely affect goblet cells, mucus permeability and microbiome composition, which were previously linked to UC [10,11]. It may be described as a low-protein, high-fiber, low-fat diet that also excludes additives. The following principles guiding food exclusion and addition included decreased exposure to sulfated amino acids (SAAs) [22,23,24], total protein [12,25,26], heme [27], animal fat [23,24], saturated and polyunsaturated fat [28], and food additives [29], with exposure to tryptophan [30,31] and natural sources of pectin and resistant starch [13,27,32,33,34]. The term “exclusion diet” was used, as the main principle of the diet is an exclusion of these foods’ components, but some other foods were added. The diet was designed with mandatory, allowed and disallowed foods. The first-phase diet is rich in fruit and vegetables, and includes mandatory foods, primarily fruits and vegetables. There are allowed foods that can be consumed without limitation, such as rice and potatoes; foods with prescribed amounts, such as chicken, eggs, yoghurt and pasta; and disallowed foods, such as red meat and processed foods. The diet also reduces sugar and fructose intake from sources other than fruits. The second phase at weeks 7–12 is more permissive, with more options of fruits and vegetables, and additions to the prescribed amounts of grain products and certain pulses. The patients were instructed on the use of the diet and were provided with recipes and a dietary support system. A UCED day sample menu is presented in Supplementary Table S1.

### 2.3. Inclusion and Exclusion Criteria

The inclusion and exclusion criteria were defined based on our initial experience, showing efficacy in cases of mild–moderate UC. Our goal was to target the appropriate UC population for a pilot study that would benefit most from a novel induction dietary therapy as an induction therapy and to test its feasibility and efficacy. The inclusion criteria included informed consent; established diagnosis of UC; age between 8 and 19 years; mild to moderate active disease defined as 10 ≤ PUCAI ≤ 45; and stable medication (IMM/5ASA) use for the past 6 weeks or no medication. The exclusion criteria included any proven current infection, such as a positive stool culture, a parasite, or positivity for *Clostridioides difficile* toxin; antibiotic or steroid use in the past 2 weeks, with the exception of patients stopping steroids at enrolment; current or past use of biologics; PUCAI > 45; acute severe UC (PUCAI ≥ 65) in the previous 12 months; a current extraintestinal manifestation of UC; and primary sclerosing cholangitis or liver disease, and pregnancy or an allergy to one of the antibiotics excluded patients from entering the antibiotic arm but not the diet.

### 2.4. Data Collection and Dietary Assessment

The patients were seen at baseline and weeks 3, 6 and 12. At week 2, a phone call visit was performed to assess the PUCAI and dietary compliance. At each visit, a PUCAI score was recorded. A clinical response was defined as a decrease in PUCAI score of at least 10 points, and clinical remission was defined as PUCAI < 10. The patients were asked to provide stool samples for fecal calprotectin (FC) at weeks 0, 3 and 6, which were analyzed locally at each participating center. We performed 24 h recall via a dietitian at weeks 0, 3 and 6. The patients were asked to record the foods and beverages and the consumed amounts in a food diary (FD) over a 3-day period (weekend and 2 weekdays) at week 3. A modified diet-adherence questionnaire [16] was completed on weeks 3 and 6. High diet adherence was determined by finding high adherence on the questionnaire and by the dietitian’s assessment based on direct questioning. Poor compliance was defined by having low compliance in any assessment.

### 2.5. Statistical Analysis

Continuous variables were evaluated for distribution normality and are reported as medians (interquartile ranges, IQRs) or means (standard deviations, SDs) as appropriate. Nominal variables are summarized as frequencies and are presented as *n* (%). The primary end point of the proportion of patients in remission at week 6 was analyzed according to the intention-to-treat (ITT) paradigm. A pairwise comparison of the PUCAI at week 0 versus week 6/week 12 was analyzed using the Wilcoxon signed rank test and used according to the last-observation-carried-forward (LOCF) approach. A pairwise comparison of the FC at week 0 versus week 6 was analyzed using the Wilcoxon signed rank test and was performed only for subjects with parameters at both time points. The food macronutrient and micronutrient daily intake was based on the food records at baseline before starting the diet and during the UCED phase 1 at week 6 or week 3, and was compared using the *t*-test for paired samples or the Wilcoxon signed-rank test as appropriate. Only patients with both week 0 and week 3/6 diet records were entered into the nutritional analysis. All the statistical analyses were performed using the SPSS version 27 statistical analysis software (IBM, Endicott, NY, USA). All the tests were two sided and were considered to be significant at *p* values < 0.05.

## 3. Results

### 3.1. Study Population

Thirty-two patients were screened between November 2014 and November 2020; eight patients were excluded (Supplementary Figure S1). Twenty-four UCED treatment courses were given to 23 eligible, consenting patients and were included in the final analysis. Significant delays in enrollment were encountered, as some of the collaborators left their institutions during the trial and there was significant delay in the ethical approval in other institutions. One patient received a second course of the UCED after a relapse two years later. The mean age of the included patients was 15.3 ± 2.9 years, with a mean disease duration of 1.4 ± 1.4 years. Demographic data are presented in Table 1. The majority had a moderate disease severity and had failed 5ASA, one patient was newly diagnosed with treatment-naïve UC, and one was coming off a course of steroids and flared.

### 3.2. Response to UCED Exclusively by Week 6

**Figure 2:**
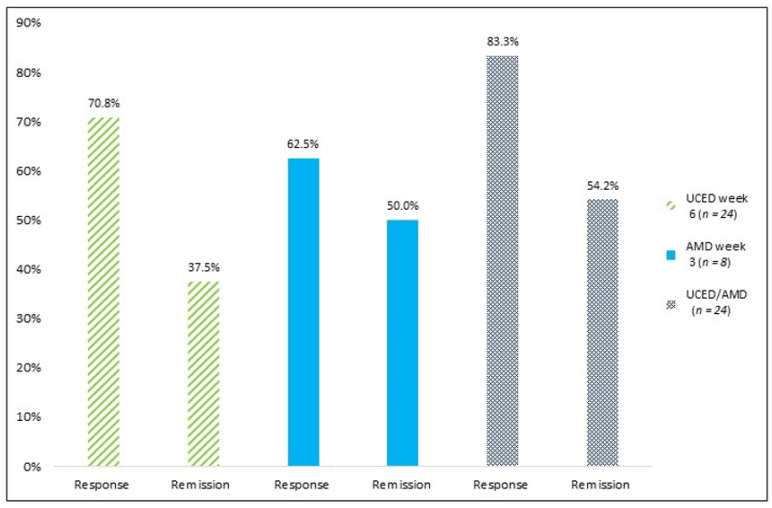
Response and remission rates according to intention-to-treat analysis with UC exclusion diet (UCED) as sole intervention; amoxycillin, metronidazole and doxycycline (AMD) antibiotic treatment; and either UCED or the AMD antibiotic treatment.

**Figure 3:**
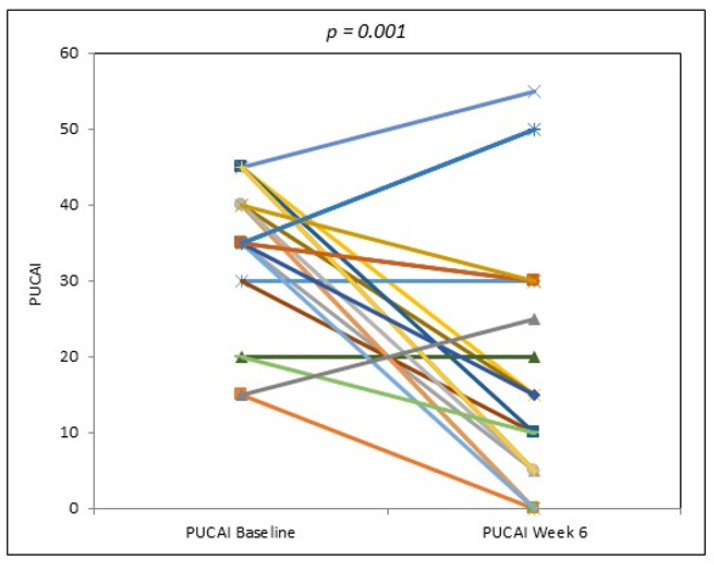
Change in PUCAI with UC exclusion diet between baseline and week 6, including all patients in a last-observation-carried-forward analysis.

Clinical responses to UCED were achieved in 17/24 (70.8%) patients by week 6, and 9/24 (37.5%) had ITT clinical remissions at week 6 with the UCED (Figure 2). One patient entered into a second remission after receiving another course of UCED, two years after the initial response. For the 23 patients, 8/23 (34.8%) had ITT clinical remissions at week 6 with the UCED. Withdrawals in remission were imputed as non-remission in the ITT analysis. The median PUCAI decreased from 35 (30–40) at baseline to 12.5 (5–30) at week 6, and the mean PUCAI decreased from 34.0 ± 10 to 17.3 ± 16.9 (*p* = 0.001) according to the ITT analysis including all the patients in a LOCF analysis (Figure 3). There were no differences in the baseline median PUCAI score, baseline FC levels and disease extent between patients who entered remission at week 6 versus treatment failures. The FC level did not vary between the recruited centers. Six patients withdrew by week 3: two noncompliant patients (one response and one remission) and four patients who required additional therapy (Supplementary Figure S1). FC results were available for 18 patients. The median FC remained unchanged from week 0 to week 3 ((818 (630.0–1880.0) μg/g and 968.0 (272.0–1798.4) μg/g, respectively (*p* = 0.76)) and declined from week 3 to week 6 of the diet ((968.0 (272.0–1880.0) μg/g to 592.0 (140.7–1555.0) μg/g, respectively (*p* = 0.41)), corresponding to a 49% reduction from week 3 to week 6; the decline between week 0 and week 6 was not significant (*p* = 0.11). Among five patients who achieved remission at week 6 with baseline and week 6 FC, the median FC level decreased from 630 (IQR, 332–1586) μg/g at week 0 to 230 (75–1298) μg/g at week 6 (*p* = 0.14).

Among the patients who were in clinical remission at week 6, seven were slow responders and achieved clinical remission only at week 6; the other two patients achieved remission at week 2 and week 3. Two patients achieved remission at week 2 and 3, respectively, but developed recurrence of mild symptoms by week 6 and were considered failures by ITT. One patient developed a *Shigella* infection during the trial that led to symptoms despite a marked reduction in FC from 1167 to 111 at week 6. Among the patients whose PUCAIs increased from baseline to week 6, it was interesting to see that two of the three patients had proctitis with moderate disease of about 1-year duration and a family history of IBD.

### 3.3. Sustained Remission with UCED at Week 12

Six out of nine patients (66%) maintained remission through week 12 without additional therapy; thus, clinical remission was observed in 6/24 (25%) at week 12 based on ITT analysis. One patient withdrew despite remission and stopped the diet; two patients experienced relapses between weeks 7 and 12: one patient developed mild intermittent bleeding without other symptoms (PUCAI: 10), and one patient developed a mild relapse. The median PUCAI decreased from 35 (30–40) at baseline to 15 (5–30) at week 12 (*p* = 0.002) according to ITT analysis including all the patients in a LOCF analysis.

### 3.4. Response to ADM after UCED Failure

Eight patients received treatment with antibiotics after failing the diet; 4/8 (50.0%) subsequently entered remission (Figure 2). Thus, in total, 13/24 (54.2%) patients obtained remission; of those, nine patients were on the diet alone, and four, on a sequential diet and antibiotic therapy as induction therapy.

### 3.5. Tolerance and Adherence

Three patients stopped the diet (two stopped despite good responses at week 3—a PUCAI of 0 and PUCAI of 10); thus, intolerance occurred in 3/24 (12.5%). The adherence to the diet was available for 22/24 (91.7%) patients at week 3; 19 (86.4%) patients had high compliance, 2 had fair compliance (9.1%) and 1 (4.5%) had poor compliance. Data for adherence at week 6 were available for 15 patients among the 16 patients who reached this week; all were highly compliant.

### 3.6. Nutritional Outcomes

**Figure 4:**
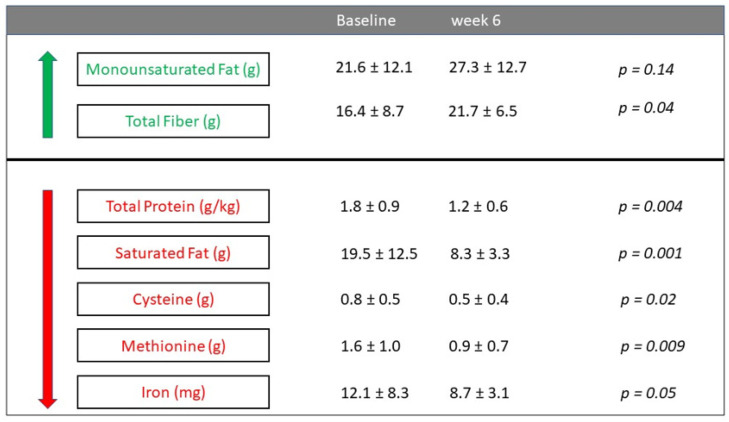
Dietary intake of macronutrients and micronutrient targets for UCED at baseline and after diet treatment: total protein (g/kg, *n* = 20), saturated fat (g, *n* = 19), monounsaturated fat (g, *n* = 19), fiber (g, *n* = 20), cysteine (g, *n* = 15), methionine (g, *n* = 15), and iron (mg, *n* = 19).

As the diet was designed to decrease animal saturated fat, total protein, SAAs and heme while providing fiber, we analyzed dietary intake before and after UCED. The analysis of dietary intake showed a clinically relevant decrease in total energy per day, as at baseline, the mean daily energy intake was 42.3 ± 25.2 kcal/kg/day, versus 32.7 ± 14.2 at week 6 (*p* = 0.06). In addition to energy intake reduction, the median weight decreased from 62 (57–65) to 59 kg (52–63) after 6 weeks of the diet (*p* = 0.02), with a mean weight loss of 0.4 ± 0.3 kg per week. Treatment with UCED was accompanied by a significant decrease in total protein, SAAs, saturated fat and iron, while there was a significant increase in total fiber consumption per day (Figure 4).

### 3.7. Safety

During the UCED treatment, eight patients had adverse events. Three patients had worsening of disease at week 3, two patients developed constipation, one patient lost weight during the first six weeks, and one patient developed a fever unrelated to the disease. Among the patients who received AMD, three patients had worsening of the disease, one patient had metronidazole intolerance with diarrhea, and one patient developed pneumonia one week after stopping the antibiotics.

## 4. Discussion

In this pilot study, we evaluated two therapies targeting the microbiome sequentially. The first intervention was a novel diet targeting the intestinal epithelium, goblet cells and innate immune system, in addition to the microbiota composition. The second intervention, used only in dietary-failure patients, was an established antibiotic protocol [20,21] studied in adults but never prospectively evaluated in children. The main purpose of this study was, first, to evaluate the feasibility of this specific dietary intervention, in order to improve the design and adherence, prior to starting an interventional randomized controlled trial. In light of this study’s outcomes, we will test the superiority of the UCED when administered together with a 5ASA regimen, compared to 5ASA alone, in pediatric patients with mild–moderate UC in a randomized control trial.

We demonstrated clinical responses in 70% of the patients with UCED at week 6 and clinical remission in 37.5% of the patients at week 6 by ITT analysis. This was accompanied by a decline in FC, primarily after week 3, which did not show statistical significance, likely due to the small sample size. The FC at week 6 was available for 5/9 patients in remission before any change in therapy; there was a decrease in the median FC among these patients from 630 (IQR, 332–1586) μg/g at week 0 to 230 (75–1298) μg/g at week 6 (*p* = 0.14).

Furthermore, 50% of those who failed to obtain remission with the diet entered remission after adding a 14-day course of AMD. Thus, over 50% of the patients obtained remission without immune suppression; of those, nine patients did so on diet alone and four, on a sequential diet and antibiotic therapy as induction therapy. We chose this sequential design in order to gain insight into the independent effect of each treatment and to provide pilot data in order to proceed to randomized controlled trials in the future based on the outcomes.

The UCED diet was designed to minimize the impact of dietary components that may adversely affect goblet cells, mucus permeability and microbiome composition, which were previously linked to UC. The UCED was designed to decrease protein, SAAs and saturated fatty acids while providing fiber as a substrate for SCFA and to prevent fiber deprivation, which may deplete the mucus layer. We were able to demonstrate that the intake of these components was, in fact, reduced among our patients, while the fiber intake significantly increased (Figure 4). Van der Post et al. have established that a permeable mucus layer may be an early event in UC [10,11]. Microbial SCFA production is essential for providing fuel for epithelial cells and affects the regulation of the immune system by inducing the regulation of T-regulatory cells [35]. A high-fat diet and maltodextrin have been shown to negatively affect goblet cells [24,36,37,38]. Epithelial damage is also a hallmark of UC, and a high-fat diet and high-protein diet may negatively affect epithelial cells [37,39]. Permeable mucus has been associated with *Proteobacteria* expansion [40], and a high-fat diet has been shown to be associated with *Proteobacteria* and *Enterobacteriaceae* expansion [39,40].

Another factor that may affect the mucus layer is fiber deprivation [34]; certain fibers such as pectins might induce more viscous mucus and have an anti-inflammatory effect [13,41]. Finally, high levels of hydrogen sulfide may have a toxic effect on epithelial cells and can cause a breakdown of the mucin network [22]. Substrates for hydrogen-sulfide production are predominantly derived from SAAs [42], while fruits and vegetables are sources of short-chain fatty acids, which regulate the production of protein metabolites and maintain tight junctions [43,44]. We used these principles to design this diet, and the results of this pilot study have led us to launch a randomized control trial (NIH NCT03980405). At this juncture, we cannot be certain which components that were included or excluded were responsible for the clinical effect or what the effect upon the microbiome was.

There are a few clinical studies that have suggested a link between diet and UC, but the data are conflicting. A prospective interventional crossover study in 18 UC adult patients showed that a low-fat, high-fiber diet decreased markers of inflammation and reduced intestinal dysbiosis [45]. A large prospective UC cohort followed from remission suggested that relapse was associated with the intake of the saturated fatty acid myristic acid, found primarily in grain-fed beef and dairy [28,46]. The benefit of plant-based diets (PBD) in UC patients was demonstrated by Chiba M. et al. to contribute to preventing relapse at one-year follow-up in UC patients; therapy incorporating a PBD was shown to induce remission in about one-third of patients with mild UC [47]. However, a large Swiss prospective cohort study showed that vegetarians had no advantage over omnivores with regard to disease activity, hospitalizations, complications or surgery with UC [48].

Most of the patients in our study tolerated the diet well, and only 12% discontinued the diet. Interestingly, two of these patients were in remission at week 3 and had a mild increase in symptoms by week six; one patient who had responded very well to the diet developed a *Shigella* infection during the trial that led to symptoms, despite a marked reduction in FC from 1167 to 111 at week 6; these four patients were considered failures in the ITT analysis.

We observed that the majority of the patients responded to the diet only between weeks 3 and 6; this is supported by the FC data, which did not show a decline between baseline and week 3 but showed a 49% decline between weeks 3 and 6. This differs from the response to exclusive enteral nutrition or the Crohn’s disease exclusion diet, in which the response was rapid and the majority of patients achieved remission during the first 3 weeks of dietary therapy [49]. Despite the decline, the median FC remained high at week 6; larger studies are required to detect the impact of diet on gut healing, including performing colonoscopies.

Antibiotics may be a double-edged sword in IBD. Antibiotics may increase dysbiosis and increase the translocation of bacteria [50] but, on the other hand, may be effective in refractory patients [7,20,21], and there is an interaction between diet and antibiotics with regard to inflammation in rodent models (a high-fat diet may increase antibiotic-induced dysbiosis) [39]. To date, the utility of the antibiotic combination we used in the trial has been demonstrated prospectively only for severe or steroid-dependent adult UC [7,20,21]. Here, we demonstrate, in a small cohort of diet-refractory patients, that antibiotic therapy may have benefit in achieving remission, and a prospective randomized controlled trial is currently underway to evaluate this further.

There are several limitations of this study. This was a pilot trial used to generate data and conducted as a proof of concept, to allow progress to larger trials if the data were positive. Thus, the sample size was limited. We only investigated children with mild to moderate disease; based on previous clinical experience, this group is the most likely to benefit from the combination of diet and antibiotics, and this combination might be used to avoid steroids and immunosuppressive therapy in the future. We were also hampered by the fact that not all the patients provided FC samples as requested. In addition, we saw a weight reduction after 6 weeks of the diet, which might indicate that the diet is not suitable for severe cases of UC with malnutrition. Another weakness of the study is that we did not perform colonoscopy in order to evaluate mucosal healing. However, recently, we have published a randomized controlled trial in adult patients with active refractory UC, showing that the UCED alone appeared to achieve mucosal healing versus single-donor fecal transplantation with or without diet, as mucosal healing (Mayo 0) was achieved only in the group that received the UCED (3/15, 20%) vs. 0/36 of the patients who received fecal transplantation (*p* = 0.022) [51]. However, we emphasize that, without a placebo group, caution needs to be taken in interpreting our results, as some of the response could be mediated by placebo effects. The strengths of this pilot trial were the prospective nature and use of defined criteria for inclusion and remission, as well as it being the first report of this novel diet.

In conclusion, the results of this pilot trial suggest that both diet and antibiotics may have a role for the induction of remission in mild to moderate UC in children. This needs to be explored further with a larger sample size. Randomized controlled trials are now underway with both therapies to provide more evidence for these therapies, which could facilitate the use of microbiome-targeted therapies in conjunction with other medical therapy or instead of immune suppression in the future.

## Figures and Tables

**Table 1:** Characteristics of the study patients at baseline.

Characteristic	Total (*n* = 24)
Female gender, *n* (%)	12 (50.0)
Age (years), mean (SD)	15.3 (2.9)
Disease duration (years), median (IQR)	1.0 (0.4–1.9)
Family history of IBD, *n* (%)	5 (20.8)
Fecal calprotectin, μg/g, (*n* = 18), median (IQR)	818.5 (630–1880)
CRP, mg/dL, median (IQR)	0.5 (0.3–0.5)
PUCAI	
Mean (SD)	34.0 (10.0)
Median (IQR)	35 (30–40)
Disease severity, PUCAI, *n* (%)	
Mild (10–30)	7 (29.2)
Range of mild disease	15–30
Moderate (35–45)	17 (70.8)
Range of moderate disease	35–45
Severe	0 (0)
Disease location, *n* (%)	
Pancolitis	4 (16.7)
Extensive	1 (4.2)
Left sided	14 (58.3)
Proctitis	5 (20.8)
Current treatment, *n* (%)	
5-ASA (oral or oral and topical)	20 (83.3)
None	3 (12.5)
Immunomodulators	2 (8.3)
Steroids *	1 (4.2)
Height (cm), mean (SD)	161.7 (13.0)
Weight (kg), median (IQR)	58.4 (43.8–64.9)
Weight z-score, mean (SD)	0.04 (1.3)

SD, standard deviation; IQR, interquartile range; IBD, inflammatory bowel disease; CRP, C-reactive protein; PUCAI, pediatric ulcerative colitis activity index; 5-ASA, 5-aminosalicylic acid. * Steroids were stopped at inclusion.

## Data Availability

The data underlying this article are available in the article and in its online supplementary material.

## Supplementary Materials

The following are available online at https://www.mdpi.com/article/10.3390/nu13113736/s1. Figure S1: Participant flow diagram, Table S1: A day’s sample menu for phase 1 UCED.

## Abbreviations

UC	Ulcerative Colitis
UCED	Ulcerative Colitis Exclusion Diet
PUCAI	Pediatric UC Activity Index
ITT	Intention to Treat
IBD	Inflammatory Bowel Disease
5ASA	5-Aminosalicylic Acid
IMM	Immune Modulator
FC	Fecal Calprotectin
LOCF	Last Observation Carried Forward
IQR	Interquartile Range
AMD	Amoxycillin, Metronidazole and Doxycycline
SAAs	Sulfated Amino Acids
