# Supplementary material for: A Novel UC Exclusion Diet and Antibiotics for Treatment of Mild to Moderate Pediatric Ulcerative Colitis: A Prospective Open-Label Pilot Study
Source: Nutrients. 2021 Oct 23;13(11):3736. doi: 10.3390/nu13113736 (PMC8622458; doi:10.3390/nu13113736)
Supplement: Supplementary file 1 [file nutrients-13-03736-s001.zip › nutrients-1399283-supplementary.pdf]

Supplementary materials

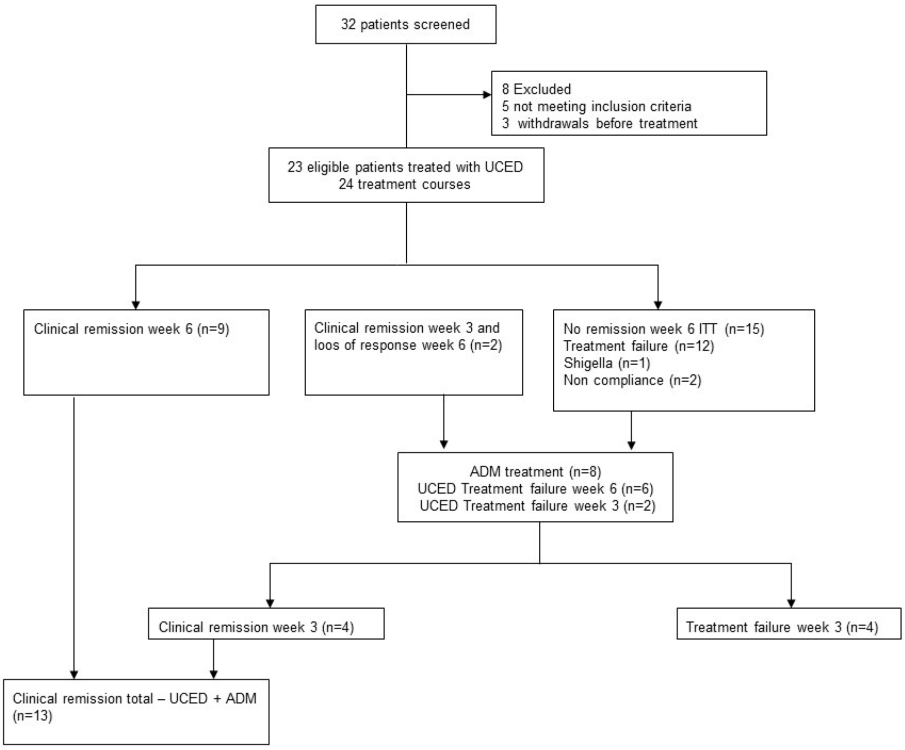

Supplementary Figure S1. Participant flow diagram

Supplementary Table S1. A day's sample menu of phase 1 UCED

Breakfast	Oatmeal with fruits or 2-3 home made rice flour crepes with fruit/or 1 + freshly squeezed orange juice
Morning snack	1 apple and rice flour tortilla with avocado
Lunch	1 baked potato (cooked and cooled before used) or cooked green beans
	Fresh stir-fried chicken breast (200 gr) or chicken balls with pasta and tomatoes sauce
	Mixed vegetable salad
Afternoon snack	1 banana or homemade potato chips (2 potatoes) or fruit sorbet with almonds
Dinner	Spring rolls with vegetables (1 zucchini, ½ red pepper) and small lettuce and tomatoes salad or quinoa salad
Night snack	1 banana or fruit salad or rice flour pancakes
